# Prevalence of Enteric Fever Pathogens Isolated from Blood Culture at a Tertiary Care Centre

**DOI:** 10.31729/jnma.5748

**Published:** 2021-03-31

**Authors:** Ruchee Manandhar, Bijendra Raj Raghubanshi, Sweekrity Neupane, Rajni Lama

**Affiliations:** 1 Department of Microbiology, KIST Medical College and Teaching Hospital, Lalitpur, Nepal

**Keywords:** *enteric fever*, *multidrug resistant*, *Salmonella*, *sensitivity test*, *typhoid*

## Abstract

**Introduction:**

Typhoid fever and paratyphoid fever commonly called as enteric fever is a life-threatening illness caused by *Salmonella* serotype Typhi and *Salmonella* serotype Paratyphi, respectively. It is a major public health issue in underdeveloped and developing countries. The aim of the study is to find out the prevalence of enteric fever pathogens in blood culture of patients attending a tertiary care centre.

**Methods:**

A descriptive cross-sectional study was conducted in 3483 blood samples of patients attending a tertiary care centre, with the history and symptoms suspicious of enteric fever during one year period from mid-September 2019 to mid-September 2020 after ethical approval from the institutional review committee. Isolates were identified by standard microbiological methods and tested for in vitro antibiotic susceptibility by modified kirby-bauer disc diffusion method. The obtained data was entered and analyzed in WHONET 5.6 program, point estimate at 95% was calculated along with frequency and proportion for binary data.

**Results:**

In our study, enteric fever pathogens were isolated from 18 (0.51%) blood samples. Out of which, *Salmonella* Paratyphi A was isolated from 10 (8.19%) and *Salmonella* Typhi was isolated from 8 (6.55%) blood samples. Other serotypes were not isolated. Antimicrobial susceptibility test showed that salmonella species that was isolated were sensitive to most of the drugs.

**Conclusions:**

Prevalence of enteric fever pathogens was lesser compared to other studies. Varying degrees of antibiotic resistance among isolated enteric fever pathogens necessitates continuous surveillance of the susceptibility patterns. Prudent use of antimicrobials, active infection control practices and stringent antibiotic policy should be implemented to prevent emergence of antibiotic resistance and future outbreaks.

## INTRODUCTION

Enteric fever is a life-threatening illnesses consisting of typhoid fever and paratyphoid fever caused by Salmonella serotype Typhi and Salmonella serotype Paratyphi respectively.^1,2^ Enteric fever is a major public health concern in many developing countries.^3,4^ Increased mortality and morbidity is attributed to rapid population growth, unplanned urbanization and improper waste disposal and water supply.^5^

Blood culture is most relevant in the first to third week from the onset of the illness. Isolation, prompt identification and accurate antibiotic sensitivity test helps in timely management of the illness.^6^

Chloramphenicol, ampicillin and cotrimoxazole had been the first line drugs for management of enteric fevers. However, strains that are resistant to these commonly prescribed antibiotics have emerged. Cephalosporins and macrolides are nowadays the therapeutic choices for enteric fever, resistance to which, has challenged developing countries like Nepal.^7,8^ Therefore, surveillance of sensitivity patterns guides clinical management at the local level.^9^

The aim of the study is to find out the prevalence of enteric fever pathogens in blood culture of patients attending KIST Medical College & Teaching Hospital.

## METHODS

This descriptive cross-sectional study was conducted from 15 September 2019 to 15 September 2020 in department of microbiology, KIST Medical College and Teaching Hospital, Lalitpur, Nepal after obtaining ethical clearance from the Institutional Review Committee of KISTMCTH IRC NO: 2076/77/12).

A total of 3483 blood samples from patients attending different departments of KISTMCTH, including the wards and outpatient departments with the history and symptoms suspicious of enteric fever were studied. Unlabeled and mislabeled blood samples, inadequate volume of samples and blood samples received after 24 hours of collection were excluded from the study. Sample Size was calculated using the formula:

$$\begin{array}{ll} \text{n} & ={\text{Z}}^{\text{2}}\times \text{p}\times \text{q}/{\text{e}}^{\text{2}}\\ & ={\left(1.96\right)}^{2}\times (0.054)\times (1-0.054)/{\left(0.03\right)}^{\text{2}}\\ & =218 \end{array}$$

Where,

n = required sample size,
Z = 1.96 at 95% Confidence Interval
p = prevalence of enteric pathogens, 5.4%^10^
q = 1-p
e = margin of error, 3%

The minimum sample size calculated was 218 and the total blood samples of 3483 were collected.

All nurses and lab technicians involved in blood collection were instructed on proper collection of blood sample in order to produce minimum contamination. For adult patients, 5-10 ml of blood sample was collected whereas 1-5 ml blood sample was collected for children. Blood sample was then inoculated into the brain heart infusion broth in the blood culture bottle. The culture bottles were properly labeled and incubated at 37°C. Each sample was subcultured after 24 hours of aerobic incubation on to blood agar, chocolate agar and MacConkey agar respectively. The preliminary report of no growth was issued when no bacteria were isolated after 72 hours. Bacterial identification was based on standard microbiological methods. Salmonella species produces large, circular, low convex, smooth, pale colonies on blood agar. Non lactose fermenting colonies are produced in MacConkey agar. Further speciation was done by biochemical tests. Salmonella species ferment glucose but not lactose and sucrose in triple sugar iron agar. All salmonella species produce hydrogen sulphide except for *Salmonella* Paratyphi A. All salmonella species are motile except *Salmonella gallinarum* and *Salmonella pullorum.* Indole is not produced. Citrate test is positive. Urea is not hydrolysed. They are methyl red test positive and vogues proskaeur test negative. When the biochemical test of the isolate was characteristics of salmonella, serological agglutination test on a suspension of about 10^10^ bacteria per millilitre were carried out, first using polyvalent sera and if positive using monovalent sera as well, in the following order.^11^

- Salmonella polyvalent O sera
- Individual salmonella O group sera
- Single factor H sera

Antimicrobial susceptibility was determined by modified kirby bauer disk diffusion method following the criteria designed by the Clinical and Laboratory Standards Institute (CLSI 2011). The obtained data was entered and analyzed in WHONET 5.6 program.

Potential bias are selection bias as antibiotic status of the patient is not known.

## RESULTS

**Figure 1.**
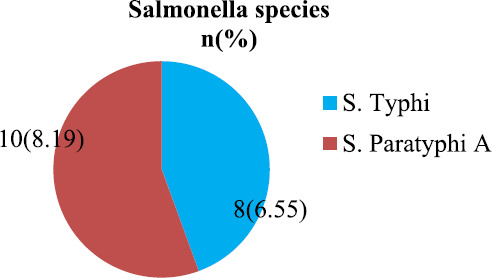
Salmonella species isolated.

Enteric fever pathogens were isolated from 18 (0.51%) out of 3483 blood samples. Growth of non-enteric fever pathogens was observed in 122 (3.5%) blood samples. Out of 18 blood samples where salmonella species was isolated, *Salmonella* Paratyphi A was isolated from 10 (8.19%) and *Salmonella* Typhi was isolated from 8 (6.55%) blood samples (Figure 1). Other serotypes were not isolated. There was no growth in 3361 (96.49%) samples.

Other non-enteric fever pathogens that were isolated included *Staphylococcus aureus* 33 (27%), Acinetobacter species 16 (13.11%), *Klebsiella pneumoniae* 15 (12.29%), *Escherichia coli* 13 ( 10.6%), Enterococcus species 8 (6.5%), Enterobacter species 5 (4.09%), Citrobacter species 5 (4.09%), coagulase negative *Staphylococcus* 3 (2.45%), Streptococcus species 2 (1.63%), *Pseudomonas aeurigenosa* 1 (0.81%), *Klebsiella oxytoca* 1 (0.81%). Fungal growth was obtained in 2 blood samples. i.e. *Candida albicans* 1 (0.81%) and candida non albicans species 1 (0.81%).

**Figure 2.**
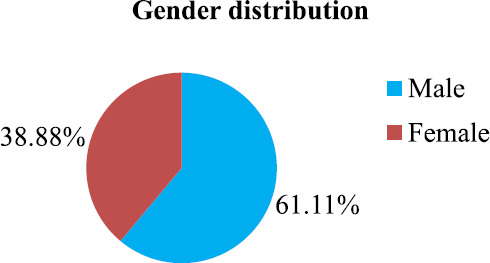
Gender distribution of isolated salmonella species.

Out of 18 samples that revealed the growth of enteric fever pathogen, 11 (61.11%) belonged to males and 7 (38.88%) belonged to females (Figure 2).

Fifteen (77 %) of samples where growth of *salmonella* species was observed belonged to the age group below 30 years (Table 1).

**Table 1:** Agewise distribution of the isolated Salmonella species.

S.N.	Age group	Number of salmonella species isolated n (%)
1.	0-10 years	3 (16.6)
2.	11-20 years	6 (33.3)
3.	21-30 years	6 (33.3)
4.	31-40 years	1 (5.5)
5.	41-50 years	1(5.5)
6.	51-60 years	0 (0)
7.	Above 60 years	1 (5.5)
	T	18 (100)

Most of the enteric fever pathogen were isolated in the month of September, 5 (27.7%) and August, 5 (27.7%)

Salmonella species were tested against ampicillin, cotrimoxazole, ciprofloxacin, chloramphenicol, ceftriaxone, gentamicin, ofloxacin, ceftazidime, cefotaxime, cefepime, amikacin, amoycillin clavulinic acid and imipenem. Blood samples were not processed for presence of anaerobic bacteria, viruses or parasites.

Antimicrobial susceptibility test showed that salmonella species that were isolated was sensitive to most of the drugs. Multidrug resistant salmonella species (resistant to all three first-line antibiotics which include chloramphenicol, ampicillin, and trimethoprim-sulfamethoxazole)^8^ were not isolated. Ciprofloxacin was the most resistant drug showing resistance in 7 (38.88%) cases. Among the enteric fever pathogen, *Salmonella* Paratyphi A and Salmonella Typhi both were most resistant to ciprofloxacin i.e. 4 (40%) and 3 (37.5%) respectively. Only 3 (16%) of salmonella species were resistant to cefotaxime. Only 1 (10%) salmonella species *(Salmonella* Paratyphi A) was resistant to ampicillin. All salmonella species were sensitive to chloramphenicol (Table 2).

**Table 2:** Antibiotic resistance pattern of isolated salmonella species.

S. No.	Name of the isolates	No. of solates	Ampicillin n (%)	Cotrimoxazole n (%)	Ciprofloxacin n (%)	Chloramphenicol n (%)	Ceftriaxone n (%)	Gentamicin n (%)	Ofloxacin n (%)	Ceftazidime n (%)	Cefotaxime n (%)	Cefepime n (%)	Amikacin n (%)	Amoxycillin clavulinic acid n (%)
1.	*Salmonella paratyphi A*	10 (55.5)	1 (10)	0 (0)	4 (40)	0 (0)	0 (0)	0 (0)	0 (0)	0 (0)	3 (30)	0 (0)	0 (0)	0 (0)
2.	*Salmonella typhi*	8 (44.5)	0 (0)	0 (0)	3 (37.5)	0 (0)	0 (0)	0 (0)	0 (0)	0 (0)	0 (0)	0 (0)	0 (0)	0 (0)
	Total	18	1 (5.5)	0 (0)	7 (38.8)	0 (0)	0 (0)	0 (0)	0 (0)	0 (0)	3 (16.6)	0 (0)	0 (0)	0 (0)

**Figure 3.**
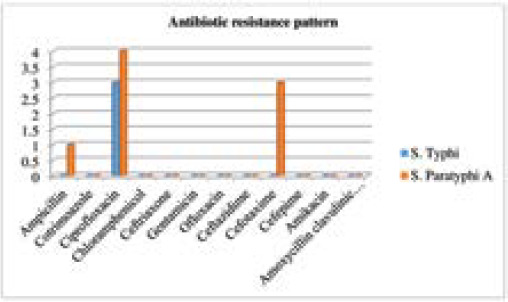
Antibiotic resistance pattern of isolated salmonella species.

## DISCUSSION

Enteric fever remains a significant health burden, especially in low- and middle-income countries.^9^ The disease has remained endemic in different areas of Nepal with outbreaks occurring time and again.^12^

Our study shows the enteric fever pathogen prevalence rate of 0.51% which was similar to studies conducted in Chitwan Medical College and National Public health Laboratory, Kathmandu which reported a prevalence rate of 0.9% and 1.02% respectively.^12,13^ However, another study conducted within Kathmandu ^10^ showed a higher prevalence rate of 5.4%. Studies conducted in India and Pakistan showed a prevalence of 4.4% and 3.4% respectively.^13,14^ This variations in the prevalence of enteric fever may result from different environmental conditions, host factors, hygiene and sanitation practices and execution of healthcare and education programmes within communities of different geographical areas. The comparatively lower isolation rate may be attributed to the COVID-19 crisis in the country when patients did not have an easier access to the healthcare facility. The lower rates of blood-culture-positive enteric fever can also be due to the use of antibiotics prior to the blood culture as well as self-medication before arrival to the hospital. ^7^ However, we did not evaluate the prior antibiotic consumption by the patients before enrollment. Like many of the studies, we did not isolate serotypes of Salmonella species other than *S.* Typhi and *S.* Paratyphi A ^10,13,14^

Blood culture for enteric fever pathogen were found to be more positive in male than females in our study which was similar to a study conducted in India^5^ This might be due to sex-linked differences in hygiene practices, males more likely to report to hospital, at the same time more likely to contract infection due to more outdoor activities.

In our study, *Salmonella* Paratyphi A had a greater prevalence (8.19%) than *Salmonella* Typhi similar to studies conducted in Chitwan^12^ and Nepal Medical College^10^ and different to study conducted in Kathmandu and India which reported higher prevalence of *Salmonella* Typhi.^5,7^ The higher prevalence of Salmonella partayphi A indicating an increasing trend of this organism in recent years^3,15^ might be due to high degree of clinical suspicion with mild fever cases being investigated for enteric fever, changing host susceptibility, change in virulence of the organism and widespread use of vaccines and quinolones against *Salmonella* Typhi in the past decade.^5^

Salmonella isolates were more common in an age group below 30 years which correlates with the study by Sharma et al. indicating that typhoid fever is most common in pre-school and school age children.^11^ Enteric fever pathogens were isolated most in the month of September and August. August and September sees normal to above normal rainfall over large parts of the country. Water is more likely to be polluted in the wet season because the rains may wash debris and littered garbage into wells and streams used as domestic sources of water.^5^ The urban lifestyle of eating street foods and drinking water also adds up to the surge in enteric fever cases during rainy seasons.

Salmonella species that were isolated in our study were sensitive to most of the drugs similar to the study conducted in Chitwan^12^ but no multidrug resistant Salmonella species were isolated. Chloramphenicol was once considered the drug of choice for enteric fever until 1989 after which chloramphenicol resistance began reported worldwide.^7^ Since the first case of Multiple Drug Resistant (MDR) S. Typhi was reported in Nepal in 1991, the use of first line antibiotics; chloramphenicol, ampicillin and cotrimoxazole became infrequent and quinolones became the first choice for treatment in endemic areas benefited by their economicity and easy availability for oral use.^7, 16^

However, as the use of ciprofloxacin increased, instead of chloramphenicol, resistance to ciprofloxacin showed an upsurge.^17^ In our study too, Salmonella species were most resistant to ciprofloxacin (38.8%) but 100% sensitive to conventional first line antibiotics, chloramphenicol and cotrimoxazole. Only 1 out of 18 salmonella isolates was found to be resistant to ampicillin. The reemergence of chloramphenicol sensitivity attributes to discontinuation of chloramphenicol therapy that relieved the selection pressure paving the way for reemergence of salmonella strains sensitive to the drug.^17^ Cephalosporin (ceftriaxone and ceftazidime) also exhibited 100% efficacy in our study as in study conducted by Bhetwal et al. indicating its effectiveness in empirical treatment of enteric fever cases.

## CONCLUSIONS

Prevalence of enteric fever pathogens was lesser compared to other studies. Varying degrees of antibiotic resistance among isolated enteric fever pathogens necessitates continuous surveillance of the susceptibility patterns. Prudent use of antimicrobials, active infection control practices and stringent antibiotic policy should be implemented to prevent emergence of antibiotic resistance and future outbreaks. Since the study was limited to a single center and convenient sampling was done, the result of the study cannot be generalized, hence, a multicentric study with a larger sample size using random sampling and study design of higher level of evidence is recommended.
